# Cell Type-Specific Anti- and Pro-Oxidative Effects of *Punica granatum* L. Ellagitannins

**DOI:** 10.3390/membranes14100218

**Published:** 2024-10-15

**Authors:** Ewa Olchowik-Grabarek, Szymon Sekowski, Iga Mierzwinska, Izabela Zukowska, Nodira Abdulladjanova, Vadim Shlyonsky, Maria Zamaraeva

**Affiliations:** 1 Laboratory of Molecular Biophysics, Department of Microbiology and Biotechnology, Faculty of Biology, University of Bialystok, 15-245 Bialystok, Poland; ewaolch@uwb.edu.pl (E.O.-G.); s.sekowski@uwb.edu.pl (S.S.); igamierzwinska@gmail.com (I.M.); izabelalenda@op.pl (I.Z.); m.zamaraeva@uwb.edu.pl (M.Z.); 2 Institute of Bioorganic Chemistry, Academy of Sciences of the Republic of Uzbekistan, Tashkent 100125, Uzbekistan; anodira73@rambler.ru; 3 Laboratory of Physiology and Pharmacology, Faculty of Medicine, Université libre de Bruxelles, 1070 Brussels, Belgium

**Keywords:** antibacterial, anticancer, antihemolytic, ROS, membrane interaction

## Abstract

Pomegranate and its by-products contain a broad spectrum of phytochemicals, such as flavonoids, phenolic acids and tannins, having pleiotropic preventive and prophylactic properties in health disorders related to oxidative stress and microbial contamination. Here, we examined the biological effects of a pomegranate peel ellagitannins-enriched (>90%) extract, PETE. In vitro studies revealed that PETE has a strong antiradical action towards synthetic radicals and biologically relevant ROS surpassing or comparable to that of Trolox. In cellular models, it showed concentration-dependent (25–100 µg/mL) yet opposing effects depending on the cell membrane type and exposure conditions. In erythrocytes, PETE protected membrane integrity in the presence of the strong oxidant HClO and restored reduced glutathione levels to up to 85% of the control value while having much weaker acute and long-term intrinsic effects. Such protection persisted even after the removal of the extract from cells, indicating strong membrane interaction. In HeLa cancer cells, and at concentrations lower than those used for red blood cells, PETE induced robust potentiation of ROS production and mitochondrial potential dissipation, leading to autophagy-like membrane morphology changes and cell death. In *S. aureus*, the growth arrest and bacterial death in the presence of PETE (with MIC = 31.25 µg/mL and MBC = 125 µg/mL, respectively) can be linked to the tripled ROS induction by the extract in the same concentration range. This study indicates a specificity of ROS production by the pomegranate extract depending on the type of cell, the concentration of the extract and the time of incubation. This specificity witnesses a strong potential of the extract components as candidates in antioxidant and pro-oxidant therapy.

## 1. Introduction

In the era of growing bacterial antibiotic resistance and rising cases of cancer worldwide, the search for compounds that cause the death of bacteria and malignant cells via ROS production appears to be a promising research direction for novel and efficient medical treatments. The group of compounds potentially fitting the above requirements might be the secondary metabolites of plants characterized by broad pro- and antioxidant activity [1,2].

Pomegranate (*Punica granatum* L.), and mainly its fruit (juice) in particular, has been used for ages in folk medicine against diarrhea, as well as against parasitic and inflammatory disorders. Recently, the interest of scientists has focused on by-products of pomegranate (peel, seeds, pomace) containing a variety of phytochemicals, including polyphenols, which can be used as antibacterial, antiviral and anticancer prophylactics in medicine or as preservatives for food, due to their ability to alter the redox status of cells [3,4,5,6,7].

In the course of cellular metabolism of a broad spectrum of organisms from bacteria to mammalian cells, reactive oxygen and nitrogen species (RONS) are constantly produced as free radicals (O_2_^−.^,^.^OH, NO^.^) and non-radicals (^1^O_2_, H_2_O_2_). Under normal physiological conditions, the RONS play a role as mediators and regulators of cellular processes by activating or deactivating various receptors, ion channels, enzymes and other signaling molecules/pathways. RONS content in cells is regulated by the antioxidant system, which includes such enzymes as superoxide dismutase, catalase, glutathione peroxidase and glutathione reductase, together with the thioredoxin system and low molecular weight antioxidants—glutathione, uric acid, ascorbic acid and tocopherol [8,9]. A disturbance in the balance between RONS production and the antioxidant system leads to a change in the redox state of the cell, causing damage at molecular and cellular levels, which eventually results in functional impairment and even cell death. Antioxidants, in particular secondary metabolites of plants polyphenols, including tannins, are used as prophylactic and curative agents to enhance the defense properties of the organism [10].

Based on their structure, properties and route of biosynthesis, tannins are subdivided into two main groups, namely, condensed tannins (CTs) and hydrolyzable tannins (HTs), but, besides these, there is also a group of simple tannins produced by brown algae (phlorotannins), as well as a group of complex tannins having a structure of a catechin unit with glycosidic linkage to hydrolyzable tannins [11]. CTs are polymers of flavonoid units. In turn, hydrolyzable tannins are polyesters of a phenolic acid with sugar or polyols and are subdivided into gallotannins (GTs) and ellagotannins (ETs). The GTs are esters of gallic acid with a polyol core, usually glucose. The ETs are esters of hexahydroxydiphenic (HHDP) and dehydro-hexahydroxydiphenic acids, which are formed via oxidative coupling of galloyl groups of GT. In addition, ellagitannins may include gallic acid and derivatives of hexahydroxydiphenic acid as valoneoyl and chebuloyl groups. Unlike GTs, ETs can also form macrocycles, which explains their great structural diversity in nature. The polygalloyl forms of tannins differ in the type of bonding: in GTs, galloyl groups are linked by depside bonds, while, in ETs, galloyl groups are linked not only by ester bonds but also through C-C bonds [12].

Tannins, as polyphenols, exhibit high antioxidant activity. At the same time, a number of studies have shown that polyphenols can form ROS in cells under normal physiological conditions. It is believed that the pro-oxidant activity of polyphenols is realized at higher concentrations and is associated with the formation of semiquinone and quinone oxidative products [1,13,14]. Thus, polyphenols show a dual functional nature, acting as antioxidant and pro-oxidant compounds. The well-studied health-promoting effects of polyphenols are attributed to their antioxidant properties, and they are recommended as preventive/auxiliary compounds against a number of diseases associated with oxidative stress, such as cardiovascular and neurodegenerative disorders and cancer. However, less attention has been paid to the pro-oxidative properties of polyphenols and, first of all, of tannins.

A characteristic feature of tannins, unlike other polyphenols, is their high ability to precipitate proteins. An inverse dependence between astringent and oxidative activities has been established. The most active protein precipitants, such as GTs and CTs, are the least oxidatively active compared to ETs, which show the weakest protein precipitant activity [15,16]. Apparently, this dependence is related to the structural features of tannins. ET molecules and their oxidation products are less flexible due to the presence of rigid groups such as HHDP in their structure, which sterically hinders interaction with proteins [16]. At the same time, the presence of oxidatively linked galloyl groups in the molecule of ET prevents the formation of resonance-stabilized radicals and, thus, enhances their oxidative activities [15].

The aim of the present work was to study the antioxidant capacity of tannin-enriched extract from pomegranate (*Punica granatum* L.) peel (90% of which are ellagitannins) in model systems and to evaluate the cytotoxicity and ROS formation in different cell types—erythrocytes, cancer cells and bacteria.

## 2. Materials and Methods

### 2.1. Material and Chemicals

The fruits of the pomegranate variety “Kazake” were taxonomically identified in the Institute of Botany of Uzbek Academy of Sciences and were used for the isolation of tannins. The ethyl acetate extraction from fruit peel was performed according to Mavlyanov et al., 1997 [17]. Based on quantitative analysis using two-dimensional paper chromatography in solvent systems n-butanol/acetic acid/water (40:12:28) and 2% aqueous solution of acetic acid, the pomegranate ellagitannin-enriched extract (PETE) contains nine compounds: granatin A (30%), granatin B (29%), punicalin (17%), punicalagin (15%), corilagin (4%), quercimeritrin (1.7%), gallic acid (1.6%), quercetin (1.1%) and ellagic acid (0.6%).

H_2_DCF-DA (2,7-dichlorodihydrofluorescein diacetate) was purchased from Invitrogen (Thermofisher, Walthman, MA, USA), and TMRM (tetramethylrhodamine, methyl ester) from Molecular Probes (Thermofisher, Walthman, MA, USA). MEM cell culture media, PBS (phosphate-buffered saline), FBS (fetal bovine serum), trypsin and streptomycin/penicillin were from Biowest (Nuaillé, France). ABTS (2,2′-azino-bis(3-ethylbenzothiazoline-6-sulfonic acid)), DPPH (2,2-diphenyl-1-picrylhydrazyl), NADH, NBT (nitro blue tetrazolium chloride), MTT (3-(4,5-dimethylthiazol-2-yl)-2,5-diphenyltetrazolium bromide), EDTA, NPS (sodium nitroprusside), sulfanilic acid and Ellman’s reagent were from Sigma (Merck, KGaA, Darmstadt, Germany). MH broth and MH agar were from Oxoid (Basingstoke, UK). All other reagents, of the highest purity available, were purchased from POCH (Gliwice, Poland).

### 2.2. Methods

#### 2.2.1. DPPH Assay

Briefly, 995 μL of 100 µM DPPH solution in 70% ethanol and 5 μL of PETE (1–10 µg/mL) were thoroughly mixed and, after 3 min of incubation, the absorbance was measured spectrophotometrically at a wavelength of λ = 520 nm. The control probe (water) was taken as 100%. The decrease in absorbance between the control and samples was used to assess antiradical activity [18].

#### 2.2.2. ABTS Assay

The ABTS^.+^ radical cation working solution was prepared by reacting 720 μL of 2 mM ABTS in aqueous solution and 720 μL of 0.95 nM horseradish peroxidase in 50 mM KH_2_PO_4_ (pH 7.0) with 75 μL of 1 mM H_2_O_2_ in 50 mM KH_2_PO_4_ (pH 7.0). The formation of ABTS^.+^ was estimated at λ = 414 nm for 10 min. Next, 10 μL of extract solutions with different concentrations (1–10 µg/mL) was added to the reaction mixture and a decrease in absorbance at 414 nm between the control probe (water), which was set as 100%, and a probe containing extract was used for calculating the scavenging activity [18].

#### 2.2.3. Superoxide Anion Radical Assay

Briefly, superoxide radicals assay was estimated in a reaction using phenazine methosulfate—nicotinamide adenine dinucleotide (PMS/NADH) system. A total of 10 μL of 14.7 mM NADH solution was added to the spectrophotometric cuvette containing 1450 μL of PBS buffer (pH = 7.4), 18.75 μL of 10 mM EDTA and 10 μL of 9.3 mM NBT. Then, 10 μL of extract (1–10 µg/mL) was added to the probes and incubated for 5 min at room temperature. Subsequently, 1 μL of 4.95 mM PMS was added, and absorbance was measured at λ = 540 nm for 10 min against the PBS buffer. A control sample was performed analogously without the addition of the extract [19].

#### 2.2.4. Nitric Oxide Assay

Nitric oxide formation from sodium nitroprusside (NPS) in aqueous solution at physiological pH was determined by the Griess reaction. For the experiment, 2 mL of a PBS buffer solution at pH = 7.4 containing 10 mM NPS was incubated for 150 min at 25 °C with the test substance (1–50 µg/mL). Then 0.5 mL of the incubated solution was mixed with 1 mL of 0.33% sulfanilic acid in 20% glacial acetic acid and incubated for 5 min. Next, 1 mL of 0.1% naphthyl ethylene diamine dihydrochloride was added and the probes were incubated for 30 min at 25 °C. The absorbance of the probes was measured at λ = 540 nm [19].

#### 2.2.5. Preparation of Erythrocytes

Swine blood was collected at the local abattoir in the presence of 3.8% citrate as an anticoagulant at the 1:9 ratio. The citrated blood was centrifuged (837× *g*, 15 min, 4 °C), and the plasma and buffy coat were removed by aspiration. Then, the erythrocytes were washed twice with 10 mM PBS (pH = 7.4).

#### 2.2.6. Hemolysis Study

One milliliter of 1% erythrocytes suspension in 10 mM PBS (pH = 7.4) was incubated with PETE (25–100 µg/mL) for 1 h and 24 h at 37 °C. After incubation, 0.5 mL of suspension was mixed with 1 mL of PBS. To obtain 100% hemolysis, 1 mL of water was added to 0.5 mL of a control sample. The erythrocytes were centrifuged (400× *g*, 15 min). The absorbance in supernatant was measured at λ = 540 nm. The percentage of hemolysis was calculated according to the following formula:

$$\%\;of\;hemolysis=\frac{A_{s}\times 100\%}{A_{c}}$$

where *A_s_* and *A_c_* are absorbances at the test and control sample, respectively, after subtraction of the absorbance of the mixture of 0.5 mL PBS and 1 mL of water.

#### 2.2.7. Measurement of ROS in Erythrocytes

One milliliter of 1% erythrocytes suspension in 10 mM PBS (pH = 7.4) was incubated with PETE (25–100 µg/mL) for 1 h and 24 h at 37 °C. After incubation, samples were labeled with H_2_DCFH-DA probe at a concentration of 20 µM. Cells were incubated for 60 min at 37 °C. The changes in DCF fluorescence intensity were registered at λ = 480 nm excitation wavelength and λ = 530 nm emission wavelength [19].

#### 2.2.8. Detection of Reduced Glutathione

One milliliter of 1% suspension of erythrocytes was prepared in 10 mM PBS, pH = 7.4. Erythrocytes were incubated for 15 min at 37 °C in the absence or presence of PETE in the concentration range of 25–100 µg/mL. Next, samples were incubated with or without 1 mM HClO for 30 min at 37 °C. Then, 0.2 mL of 25% trichloroacetic acid was added, and the samples were centrifuged. A total of 700 µL of 0.5 M phosphate buffer (pH = 7.8) and 50 µL 5 mM Ellman’s reagent were added to 700 µL of the supernatant. After 30 min of incubation, the samples were monitored spectrophotometrically at 414 nm [20].

#### 2.2.9. Determination of Erythrocyte Oxidative Hemolysis

One milliliter of 1% suspension of erythrocytes was prepared in 10 mM PBS, pH = 7.4. Erythrocytes were incubated for 15 min at 37 °C in the presence or absence of PETE at the concentration range of 25–100 µg/mL. Next, samples were incubated with or without 1 mM HClO for 30 min at 37 °C. After incubation, 0.5 mL of suspension was mixed with 1 mL of PBS. To obtain 100% hemolysis, 1 mL of water was added to 0.5 mL of a control sample. The erythrocytes were centrifuged (400× *g*, 15 min). The absorbance of the supernatant was measured at λ = 540 nm [20].

#### 2.2.10. HeLa Cell Culture

HeLa cells were obtained from Prof. Y. Okada (Department of Cell Physiology, National Institute for Physiological Sciences, Okazaki, Japan). HeLa cells were sterile cultured (T = 37 °C, 5% of CO_2_, humidified environment) in minimum essential medium (MEM) supplemented with the heat-inactivated FBS (10%) and 677 I.U./mL penicillin and 67.7 µg/mL streptomycin. Cells were seeded on the 10 cm Petri dish at the concentration of 8 × 10^5^ cells/mL and grown to 90% confluence. For the experiments, MEM was removed and the cells were washed with sterile PBS, briefly trypsinized in trypsin/EDTA solution and detached by tapping the dish with cells in sterile PBS solution. The cell suspension was placed in a sterile falcon vial, and the number of cells was estimated using an automated cell counter (EveTM, NanoEntek, Seul, Korea).

#### 2.2.11. Measurement of HeLa Cells Viability—MTT Assay

HeLa cell viability was measured spectrophotometrically using MTT (3-(4,5-dimethylthiazol-2-yl)-2,5-diphenyltetrazolium bromide) assay. Cells (1.5·10^5^ cells/mL) were seeded on 48-well plates in MEM (supplemented with FBS and antibiotics) and incubated for 24 h. Next, the supplemented MEM was replaced by serum-free MEM (in order to eliminate the pomegranate polyphenols interaction with FBS proteins), and PETE at the final concentrations of 25 µg/mL, 50 µg/mL, 75 µg/mL and 150 µg/mL was added. Cells were incubated for the next 24 h (T = 37 °C, 5% of CO_2_, humidified environment). After incubation, MEM was removed and the cells were washed with PBS. Next, 500 µL of PBS and 10 µL of MTT (5 mg/mL in PBS) were added to each plate well. Cells with MTT were incubated for one hour at 37 °C, and the MTT solution was removed. Cells were lysed by DMSO, and the absorbance was measured using an automated plate reader at the wavelength λ = 570 nm. The absorbance of cells in the absence of PETE served as the control and was taken as 100% viable cells. Cell viability in the presence of PETE was calculated according to the equation below:

$$\%\;of\;viability=\frac{A_{s}\times 100\%}{A_{c}}$$

where *A_s_* and *A_c_* are absorbances of the test and control sample, respectively, after subtraction of the absorbance of pure DMSO.

#### 2.2.12. Analyses of HeLa Cells Morphology Changes

Changes in HeLa cell morphology in the presence of pomegranate extract were visualized using light microscopy (Olympus CKX-41, Hamburg, Germany). Probes for analyses were prepared in the same way as for MTT assay but in the 6-well plates. For the experiments, two concentrations of PETE were used (75 µg/mL and 150 µg/mL).

#### 2.2.13. Measurement of ROS—Laser Scanning Confocal Microscopy (LSCM)

Investigations of ROS generation in HeLa cells in the presence of PETE were performed using fluorescence label H_2_DCFDA (2′,7′-dichlorodihydrofluorescein diacetate), which crosses the cell membrane into cytosol, where intracellular esterase hydrolyzes it to cell-impermeant DCFH, which, in the presence of ROS, undergoes a transformation into a fluorescent derivative (DCF) [21]. Cells in the supplemented MEM were cultured for 24 h on the coverslips placed in the 6-well plates. Next, the MEM solution has been removed and replaced by serum-free MEM containing PETE at the final concentrations of 25 µg/mL and 50 µg/mL. Cells were then incubated for the next 24 h (t = 37 °C, 5% CO_2_, humidified environment). After incubation, the MEM was removed, and coverslips with cells were washed three times with PBS (supplemented with 10 mM of glucose); then, the PBS (with 10 mM glucose) containing H_2_DCFDA at the final concentration 2 µM was added to all coverslips. Cells were incubated for 45 min (in the dark) and washed four times by PBS with glucose. Such prepared cells were placed under an LSC microscope (Zeiss, Jena, Germany) and studied for the presence of ROS using fluorescence excitation and emission wavelengths: λ_exc._ = 488 nm and λ_em._ = 515 nm.

#### 2.2.14. Analysis of HeLa Cells’ Mitochondrial Potential

Studies were performed using the fluorescence label TMRM (tetramethyl-rhodamine), according to Joshi and Bakowska (2011) [22]. Briefly, cells were cultured on the coverslips and prepared in the same way as for ROS analyses. Following the incubation with PETE, cells were stained with 20 nM TMRM dissolved in PBS (with 10 mM glucose) and incubated for 45 min in the dark. Next, the LSCM images were taken at λ_exc._ = 556 nm and λ_em._ = 576 nm.

#### 2.2.15. Image Analysis

Background-subtracted cell fluorescence intensity was obtained using ImageJ-1.54 software. A minimum of 50 regions of interest (ROI), i.e., cells, from coverslips prepared on three different occasions were analyzed for each experimental condition.

#### 2.2.16. Bacterial Strain and Growth Conditions

The *S. aureus* reference strain 8325-4 obtained from Prof. Jan Oscarsson (University of Lund, Sweden) was used in this study. Bacteria were grown overnight at 37 °C in Mueller Hinton (MH) broth shaking at 200 rpm or on nutrient agar (NA) plates.

#### 2.2.17. Antimicrobial Activity

The Minimum inhibitory concentration (MIC) and Minimum bactericidal concentration (MBC) of PETE were evaluated as described earlier [23]. Briefly, the evaluation of the cell growth of *S. aureus* 8325-4 using the broth microdilution method was conducted according to the National Committee for Clinical Laboratory Standards. PETE was added to Mueller Hinton broth (MHB) to give a final concentration of 4000 µg/mL. Next, the samples were serially two-fold diluted in MHB to obtain concentrations ranging from 2000 to 0.97 µg/mL in a 96-well microtiter plate with final volumes of 100 µL. Then, 100 µL of *S. aureus* bacteria solution was injected into each well, giving a final bacteria cell concentration of 1·10^6^ colony-forming units per mL (CFU/mL). The plates were incubated at 37 °C for 24 h for bacteria. The MIC value was determined as the lowest concentration of PETE that inhibited bacterial growth, as indicated by the absence of turbidity. Minimal bactericidal concentration (MBC) was assessed as follows. Five microliters of the overnight culture from each well in the plate with tested compounds of concentration equal to and higher than the MIC value were transferred into MH agar and incubated overnight at 37 °C. The MBC value was determined as the lowest concentration of PETE, for which no bacterial growth on the nutrient agar was observed.

#### 2.2.18. Measurement of ROS in *S. aureus*

The level of reactive oxygen species in *S. aureus* in the presence of PETE was determined using an H_2_DCFH-DA fluorescent probe. Bacterial cell suspensions in PBS (OD_600_ = 0.1), PETE (5–30 μg/mL) and H_2_DCFH-DA probe at a concentration of 20 μM were added into 96-well black plates. Bacterial cells without PETE were used as a control. The samples were incubated at 37 °C for 4 h. Fluorescence values were read at excitation wavelength of λ = 488 nm and emission wavelength of λ = 535 nm. Fluorescence of 20 µM H_2_DCFH-DA alone in PBS was negligible.

#### 2.2.19. Data Analysis

All results are presented as Mean ± SD. A number of independent assays is reported in figure legends. Unless otherwise stated, five replicates were performed in each independent experiment. Statistical significance was evaluated using GraphPad QuickCalcs software (https://www.graphpad.com/quickcalcs) (accessed on 24 May 2017). One-way ANOVA followed by *t*-Student test was applied. The IC50 values were estimated by fitting the concentration–response data to the standard logistic equation using Origin 8.5.1 software (Northampton, MA, USA).

## 3. Results and Discussion

### 3.1. The Free Radical Scavenging Activity of PETE

**Figure 1:**
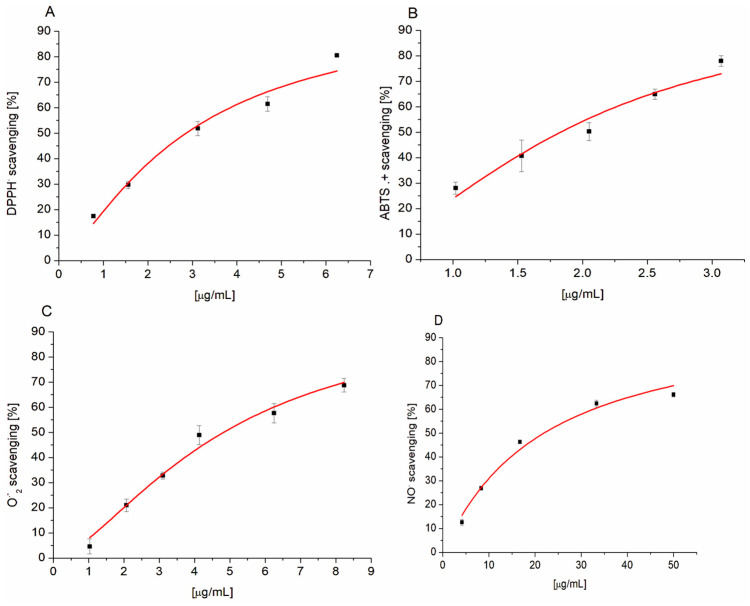
Concentration-dependent free radical scavenging activity of PETE against DPPH (**A**), ABTS^+^; (**B**), O_2_^−.^; (**C**) and NO (**D**). Lines in the graphs represent the best fit of data to the logistic equation (n = 3).

First, we studied the antiradical activity of the extract using two different synthetic radicals, namely, the charge–free radical DPPH and the cation radical ABTS^+.^. An α-tocopherol derivative, Trolox was used as a positive control to compare the antiradical activity of the extract tested. The free radical scavenging activities determined by DPPH and ABST tests were expressed as the IC_50_ value (the concentration of extract required to reduce 50% of the initial DPPH or ABTS concentration). Results are presented in Figure 1 and in Table 1.

The IC_50_ value of the extract for the scavenging ABTS^.+^ was statistically lower than the IC_50_ value of Trolox, while the difference in IC_50_ values between PETE and Trolox for the scavenging DPPH did not reach statistical significance. Nevertheless, these data suggest that the extract exhibits somewhat stronger activity against artificial radicals compared to Trolox. It should also be noted that the IC_50_ value for extract in the reaction with DPPH was 1.5 times higher compared to the respective IC_50_ value for ABTS^.+^ (*p* = 0.009). Similar values of antioxidant activity of the pomegranate peel methanol extract to those of the DPPH and ABTS were reported by Elfalleh et al. 2009 [24]. It can be speculated that the difference in the activity of ellagitannins against ABTS^.+^ and DPPH^.^ is related to the different mechanisms of action. It is known that the mechanism of antioxidant action depends on many factors, including solvent polarity, pH or the ability of antioxidants to dissociate [25]. In the case of DPPH dissolved in ethanol, the reaction with tannins may follow a one-step hydrogen atom transfer mechanism, HAT (Hydrogen Atom Transfer). On the other hand, in the reaction with the cation radical ABTS occurring in an aqueous medium, where tannins are shown to dissociate, electron transfer might be preceded by deprotonation of the OH group and so-called SPLET (Sequential Proton-Loss Electron-Transfer) may occur [25].

We also evaluated the antiradical activity of the extract against radicals formed in the organisms as a result of one-electron reduction of oxygen and enzymatic reactions, namely, superoxide anion radical and nitric oxide. The results obtained showed a concentration dependence in the range of 1–25 µg/mL with IC_50_ 4.74 and 21.91 µg/mL, respectively (Table 1). These values were two to three times lower than for sumac leaf extract containing more than 90% gallotannin [19]. Statistical comparison of the antiradical activity of the extract with Trolox showed that, in the case of NO, the effect of the extract was two times lower (*p* = 0.005) and, in the case of O_2_^−.^, it was 42 times higher (*p* = 10^−5^). Such a large difference in the IC_50_ value relative to O_2_^−.^ is not unique, a similar difference was shown previously for protocatechuic acid [26]. The importance of the high antiradical activity of the extract against O_2_^−.^, which is the first product in the ROS formation chain, should be emphasized since it characterizes the extract as a participant in the first line of defense against oxidative stress.

### 3.2. Antioxidative and Pro-Oxidative Effect of PETE in Erythrocyte Model

The next step was to evaluate the antioxidant activity of the extract in the cellular model, namely, in erythrocytes under conditions of disturbed redox homeostasis. Erythrocytes are a convenient model for studying the effect of various compounds, including polyphenols, on oxidative stress in the cell [27]. Erythrocytes, on the one hand, are cells sensitive to oxidative stress due to the high content of unsaturated fatty acids undergoing rapid degradation. On the other hand, they themselves produce a significant amount of ROS as a result of NADPH oxidase activation and hemoglobin autooxidation [28]. Oxidative stress results in structural changes in the membrane and in impaired erythrocyte function. In order to study the antioxidant properties of the extract, we used HClO as an oxidant in our experiments. It is well known that HClO penetrates into the erythrocyte membrane and may be transformed into ^.^OH and ^1^O_2_ in a nonenzymatic reaction [29]. HClO induces oxidation of protein SH groups that, in turn, causes S-S bridge formation and results in consequent changes in protein activity and intracellular glutathione degradation. The toxic effects of HClO are also connected with the formation of chloramines in reaction with the amino group of lipids, proteins and chlorohydrins resulting from lipid degeneration. Erythrocyte exposure to HClO leads to cell swelling, structural rearrangement of the membrane, pore formation, and, ultimately, hemolysis; however, it does not induce hemoglobin oxidation [30,31]. Therefore, we studied the protective effects of the tested extract on changes in glutathione content and hemolysis induced by HClO. The influence of extract on erythrocyte integrity was first evaluated. As shown in Table 2, the percentage of hemolysis and ROS content was at the control level after 1 h incubation of erythrocytes, even at the highest concentration of extract (100 µg/mL). These experiments indicate that the extract does not induce ROS formation and hemolysis in these experimental conditions. Nevertheless, prolonged incubation of erythrocytes in excess of 24 h in the presence of a higher concentration of this extract induces, per se, the appearance of ROS in erythrocytes, which, however, did not hinder their structure (Table 3). The pro-oxidant effect of PETE in the long term might be related to the accelerated aging of red blood cells during 24 h of incubation in an unnatural medium (i.e., PBS) instead of plasma. This aging is evident from the increased rate of spontaneous hemolysis at 24 h (see 0 µg/mL, Table 3 vs. Table 2, *p* = 0.018). Moreover, it is well known that aging is accompanied by a gradual decline in the antioxidant status of erythrocytes [27,28], which makes them more susceptible to high concentrations of the polyphenolic substances that are known to produce some radicals themselves [1].

**Figure 2:**
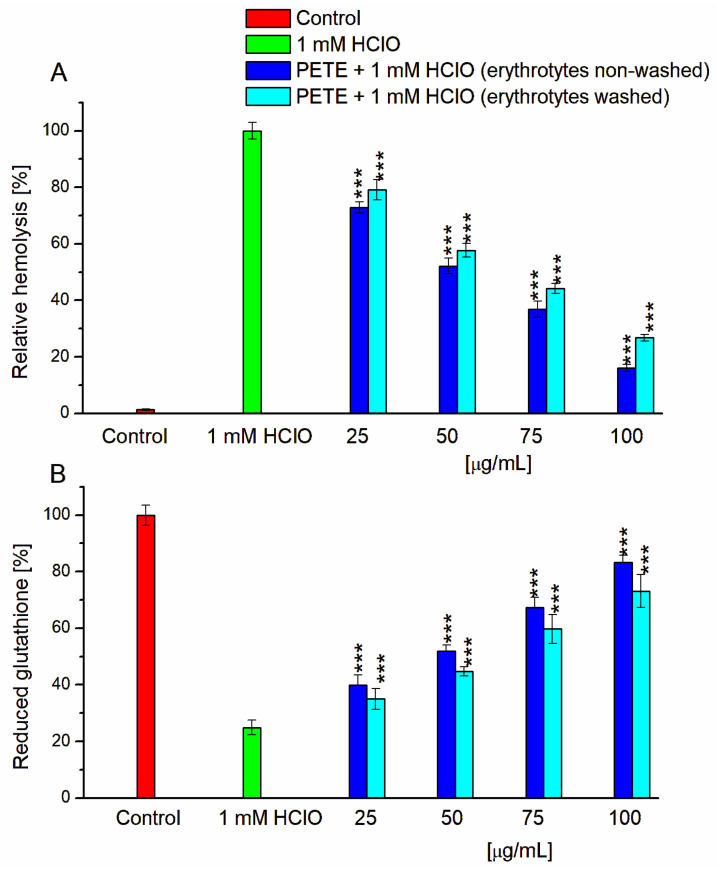
Protective effect of PETE against oxidative hemolysis (**A**) and on the level of GSH reduction (**B**) induced by 1 mM HClO. *** *p* < 0.001 vs. respective HClO (n = 5).

The incubation of erythrocytes with 1 mM HClO induced 71.63 ± 3.08% cell hemolysis, which was taken as a relative 100% in this study. As depicted in Figure 2, tannins protect erythrocytes from hemolysis in a concentration-dependent manner in the range of concentrations between 25 and 100 µg/mL. The fitting of the data to the logistic equation suggests the IC_50_ value of 48.94 ± 3.70 µg/mL. We also studied the effect of the extract on HClO-induced GSH depletion. In the absence of extract, treatment of erythrocytes with HClO resulted in a significant decrease in glutathione content (to 22.55 ± 2.16% compared to control). Preincubation of erythrocytes with extract at concentrations of 25, 50, 75 and 100 µg/mL, followed by exposure to HClO, caused a gradual increase in GSH content. Similar results were shown for CT from grape seeds (*Vitis vinifera* L.) and hydrolyzable tannins from leaves of sumac *(Rhus typhina* L.) [20].

The mechanism of antioxidant activity of the studied extract may be related both directly to their antiradical activity explored above in the reaction with DPPH and ROS and to their ability to bind iron and to change the packing density of erythrocyte membranes, preventing both the penetration of oxidants into cells and the formation of pores characteristic for HClO-induced hemolysis. However, it cannot be excluded that tannins of the extract prevent the effect of HClO directly interacting with the oxidant, reducing its effective concentration, since it has been found that tannins in the presence of HClO can undergo chlorination [32]. To test this hypothesis, we performed an experiment with erythrocytes pre-incubated with the extract and then washed, which excluded the presence of free tannins in the medium of incubation with HClO. As presented in Figure 2, the ellagitannins of the extract, in this case, also significantly protected erythrocytes from hemolysis and reduced glutathione content decrease induced by HClO. The obtained data indicate that the studied tannins bind firmly enough to erythrocyte membranes and protect them from oxidative stress. The findings are consistent with the accepted notion that the binding of tannins to erythrocyte surface proteins leads to the formation of a complex that may serve as a potent “sink” for RONS [33].

The overall obtained results indicate high antioxidant activity of the extract, 90% of which are ETs, such as granatin A, granatin B, punicalgin, punicallin and corilagin. This is consistent with previous observations showing that pomegranate juice by-products containing punicalagin and punicallin effectively block the formation of ROS in myelomonocytic HL-60 cells induced by phorbol 12-myristate-13-acetate [34]. An aqueous extract of *Cistaceae ladanifer* containing ET punicalagin and punicallin also exhibited a high level of activity in different antioxidant measurements in the same concentration range. At the same time, this extract exhibited antimicrobial activity against *S. aureus* as well as cytotoxicity against human breast cancer cells [35]. ETs also manifested anti-cancer effects against human or squamous cell carcinomas and salivary gland tumor cell lines by inducing apoptosis. Since these compounds were shown by the EPR method to be capable of producing their own radicals able to induce apoptosis, it was assumed that the anti-cancer effect was due to their pro-oxidant actions [36]. Therefore, we further studied the ability of the extract to induce ROS formation and kill bacterial (*S. aureus*) and cervical cancer (HeLa) cells.

### 3.3. Anticancer and Pro-Oxidative Activities of PETE in HeLa Cells

**Figure 3:**
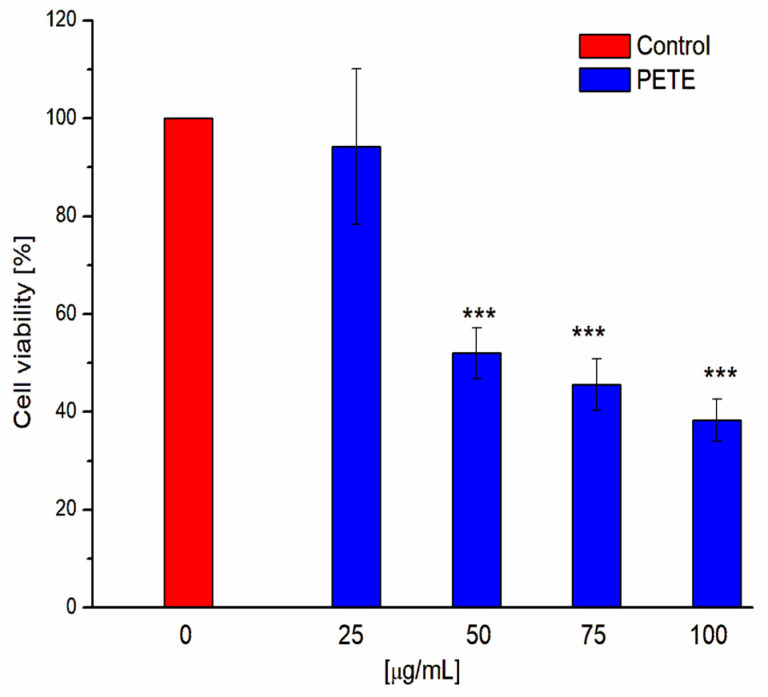
HeLa cells viability in the presence of PETE. *** *p* < 0.0005 vs. control (n = 5).

In order to investigate the influence of pomegranate extract on HeLa cell viability, we have performed the MTT assay test (Figure 3). Obtained results demonstrate that pomegranate extract exhibits concentration-dependent cytotoxicity towards HeLa cells. Our results are in good agreement with other studies on the strong anti-cancer activity of tannins. For example, Zarin et al. demonstrated that tannins isolated from Leucaena leucocephala were cytotoxic to MCF-7 breast cancer cells [37]. Sepehr et al. described that *Punica granatum* polyphenolic extract killed prostate and fibrosarcoma cancer cells [38,39]. It was also presented that different groups of tannins like proanthocyanidins, procyanidins, GTs and ETs display anti-cancer activity against lung cancer [40].

**Figure 4:**
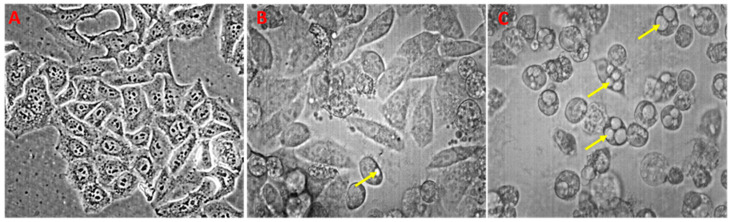
HeLa cell morphology. Control (**A**), HeLa + 75 µg/mL of PETE (**B**) and HeLa + 150 µg/mL of PETE (**C**). (Magnification 200×, yellow arrows mark vacuoles). Phase-contrast images are representative of three independent cell culture experiments.

It is known that the first step of the compounds’ influence on the cells is their interaction with the membrane, that can, in turn, induce morphology changes within the cell structure. Therefore, we studied HeLa cell morphology after treatment with PETE using light microscopy (Figure 4). Control HeLa cells (Figure 4A) present a typical shape for this cell line without any malformations in morphology. The addition of pomegranate extract induced strong and concentration-dependent changes in HeLa cell morphology with characteristic vacuolisation (yellow arrows) (Figure 4B,C). Since vacuolization is a typical characteristic of autophagy, we hypothesized that pomegranate extract induced cell mortality via this type of cell death, which is consistent with the available literature. In fact, it was described that polyphenols can induce Beclin-1 dependent (canonical) or Beclin-1 independent (non-canonical) autophagy in different cancer cells [41]. In other work, Zhao’s team presented that urolithin A (ellagitannin metabolite) induced autophagy as well as apoptosis in human colorectal cancer cells [42]. Tan et al. described that pomegranate extract possessed the ability to activate transcription factor EB (TFEB), which is responsible for the upregulation of the expression of autophagy markers in addition to having an influence on mitochondrial morphology and promoting the recruitment of autophagosomes [43].

**Figure 5:**
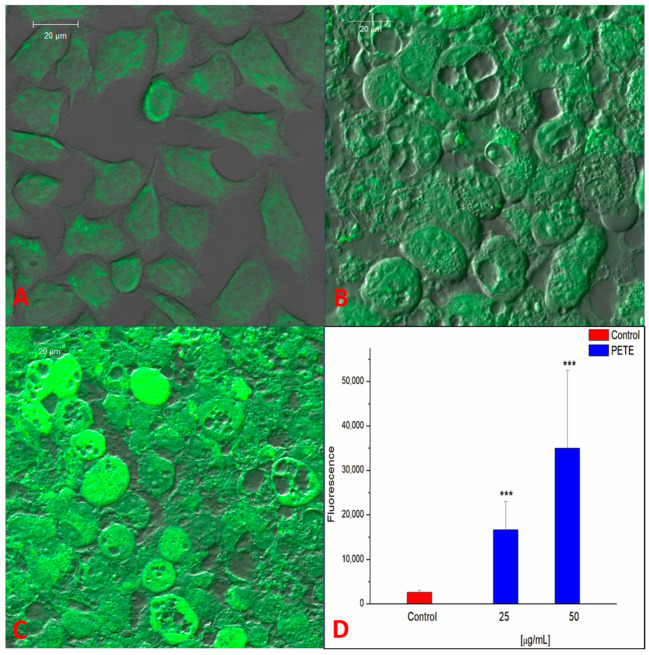
Generation of ROS in the presence of PETE in HeLa cells. LSCM images of control cell (**A**) and cells in the presence of 25 µg/mL (**B**) or 50 µg/mL PETE (**C**) are shown. (**D**) Background-subtracted fluorescence intensity of DCF label of at least 50 ROI (cells). *** *p* < 0.001. Images are representative of three independent cell culture experiments.

Based on the observations above that the pomegranate extract strongly decreased the viability and induced morphological changes in the HeLa cells, we next studied the influence of extract on the reactive oxygen species (ROS) level and mitochondrial potential (Ψ_MT_) using laser scanning confocal microscopy (LSCM). As is demonstrated in Figure 5, the pre-treatment with PETE results in a very strong and statistically significant increase in ROS in HeLa cells. The lowest used concentration (25 µg/mL) did not affect the cells’ viability; however, it drastically increased ROS generation (about 6.5 times compared to the control). For the higher PE concentrations, a further increase in ROS level was detected. Our results are in good agreement with the reports describing the potential of polyphenolic compounds to increase ROS production. For example, Elango demonstrated that gallic acid boosted ROS levels in adenocarcinoma human alveolar basal epithelial cells (A549 cells) [44]. Also, Ślęzak described that polyphenols from Cistus and Pomegranate had prooxidant activity in Chinese hamster cell lines (V79 cells) [45].

**Figure 6:**
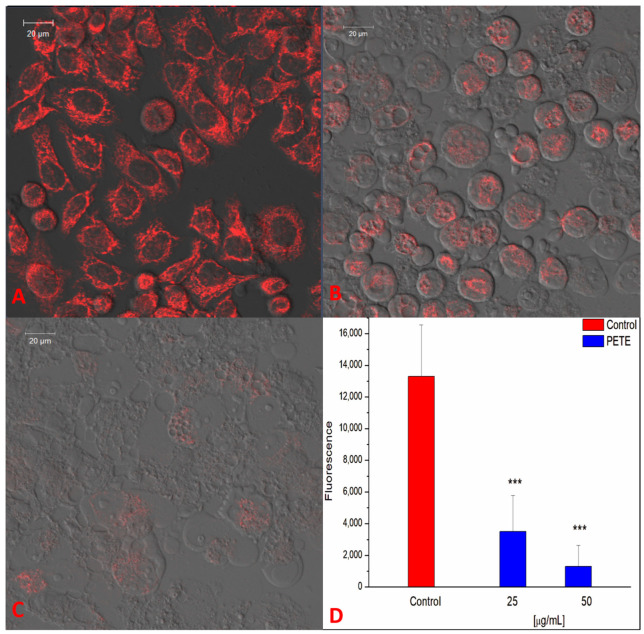
Changes in mitochondrial potential (Ψ_MT_) in the presence of PETE in HeLa cells. LSCM images of control cell (**A**), and cells in the presence of 25 µg/mL (**B**) or 50 µg/mL PETE (**C**), are shown. (**D**) Background-subtracted fluorescence intensity of TMRM dye in at least 50 ROI (cells), *** *p* < 0.001 vs. control. Images are representative of three independent cell culture experiments.

Figure 6 demonstrates that PETE strongly decreases Ψ_MT_ in a concentration-depended manner. The lowest PETE concentration, i.e., 25 µg/mL, decreased Ψ_MT_ by about 4-fold (Figure 6B,D) while 50 µg/mL reduced Ψ_MT_ by about 10-fold in comparison with the control (Figure 6C,D). Thus, it can be concluded that PE induces strong mitochondrial membrane depolarization. Mitochondrial membrane potential is the crucial factor for maintaining the cell respiratory functions mainly connected with ATP synthesis. Changes in Ψ_MT_ may result in the release of apoptotic factors and as may lead to chromatin condensation and degradation, hence inducing apoptosis [46]. Loss of mitochondrial ATP production can also induce autophagy in an mTOR/AMPK-dependent way [47]. Our results on Ψ_MT_ are consistent with the literature. For example, Wang demonstrated that tannic acid was able to reduce mitochondrial potential and ATP levels, inducing apoptosis of porcine intestinal IPEC-J2 [48]. Also, Sp et al. described that tannic acid decreased mitochondrial membrane potential and ATP concentration as well as increased the ROS level in human embryonic carcinoma cells. The generation of ROS was related to the impairment of mitochondria and, as the authors postulated, can be the result of the induction of mitochondrial ROS (mROS) [49].

Tannins exhibit a multi-target mechanism of anticancer activity inducing apoptosis or autophagy via regulation of various metabolic pathways [50]. For example, it was shown that ellagitannins (granatin A and granatin B from *Punica granatum* leaves) suppress cell lung carcinoma A549 cells by inducing apoptosis through down-regulation of microsomal PGE synthase-1 (mPGES-1) [51]. Corilagin from *Phyllanthus niruri* L. exhibited anticancer activity, inhibiting the growth of ovarian cancer cells via the TGF-β/AKT/ERK/Smad signaling pathways [52]. Tannic acid suppressed lipid metabolic pathways, induced ROS and endoplasmic reticulum (ER) stress and led to apoptosis in prostate cancer cells [53].

It should be noted that the antitumor effects of polyphenols and their action on ROS formation depend on their concentration and the exposure time, while a direct correlation between these parameters (time–concentration) is not always consistent in the literature. Early research showed that gallic acid induced HeLa cell death via apoptosis and that this effect was accompanied by ROS increase, GSH depletion and the loss of mitochondrial potential [54]. However, the same study demonstrated that high concentrations of gallic acid (100–400 µM) increased the growth of HeLa cells under conditions of a short incubation time (4 h). In a cell model of the digestive gland of mussels (*Unio tumidus*), it was shown that tannic, gallic and ellagic acids showed a pro-oxidant effect, inducing DNA damage [55]. This study reported that the effect depended on the concentration; at low concentrations of 1–5 µM and at high concentrations of 240 μM, the effect was less profound than in the range of 30–80 μM. The cytotoxicity of polyphenols of Ground Ivy (*Glechoma hederacea* L.) extract also depended on the type of cancer cells and the pro-oxidant effect, in some cases, decreased with the exposure time [56].

Two mechanisms of ROS formation in cells by polyphenols have been suggested. The first mechanism stipulates that polyphenols themselves (in the case of tannins, mainly ellagitannins) might be the source of ROS. This occurs through the formation of semiquinone radicals that are formed by one-electron oxidation of polyphenols, which depends on the extra- and intracellular environment, in particular, pH and the presence of metal ions. In the case of tannins, radicals are formed not only at a basic pH but also under the influence of peroxidases and non-enzymatic oxidants [15,57]. The second mechanism of ROS formation is related to the above-mentioned effect of polyphenols on cell metabolism and on the suppression or the activation of certain genes, leading to cell death. Namely, the pro-oxidant activity of polyphenols with variable levels of ROS induction can lead to different forms of cell death: at high levels of ROS, they induce ROS-mediated apoptosis or autophagy, and, at low levels, ROS–epigenetic cell aging [58].

It is still a matter of debate whether the increase in ROS content in cells treated with polyphenols is an inducer of apoptosis or its consequence [59]. In this respect, it should be kept in mind, once again, that low concentrations of polyphenols or short exposure times can lead to increased proliferation of cancer cells and no death at all [3]. This is because ROS induced by polyphenols may serve as signaling molecules at a certain low level, activating metabolic processes without disturbing normal cell functioning. It is not until a certain threshold of ROS concentration (characteristic for each cell type) is crossed that the growth of cancer cells is restricted, and their death is imminent.

On the other hand, polyphenols can exert anti-cancer effects not only through pro-oxidant activity by inducing ROS but also by exhibiting antioxidant activity. Cancer cells are characterized by increased metabolism, which, in turn, leads to increased formation of ROS. At the same time, ROS are known to activate the expression of antioxidant systems, which allows the cell to maintain an increased level of ROS but one that is compatible with life. In this case, antioxidant activity polyphenols lower the level of ROS, hinder malignant cell metabolism, and, thus, act in a chemopreventative manner; such an anti-cancer mechanism can be realized at the early stages of cancer development [58,60].

### 3.4. The Influence of PETE on S. aureus Growth and Viability and on Bacterial ROS Production

*Staphylococcus aureus* is a strong pathogenic Gram-positive bacterium hindering human and animal health by inducing a broad spectrum of diseases, e.g., skin diseases (folliculitis, furunculosis, dermatitis) and invasive diseases such as pneumonia, osteomyelitis and sepsis, as well as many others, including food poisoning [61]. In our studies, we used a *S. aureus* strain (8325-4) that is characterized by high toxicity due to its ability to produce α-, β-, δ- and γ-hemolysin [62]. The antimicrobial activity of the extract against strain 8325-4 was estimated in terms of MBC and MIC, the lowest concentration of the extract that results in microbial death (MBC) and the lowest concentration completely preventing the growth of bacteria (MIC). The MIC and MBC values were measured to be 31.25 µg/mL (n = 6) and 125 µg/mL (n = 6), respectively, thus indicating the antimicrobial activity of pomegranate extract constituents.

**Figure 7:**
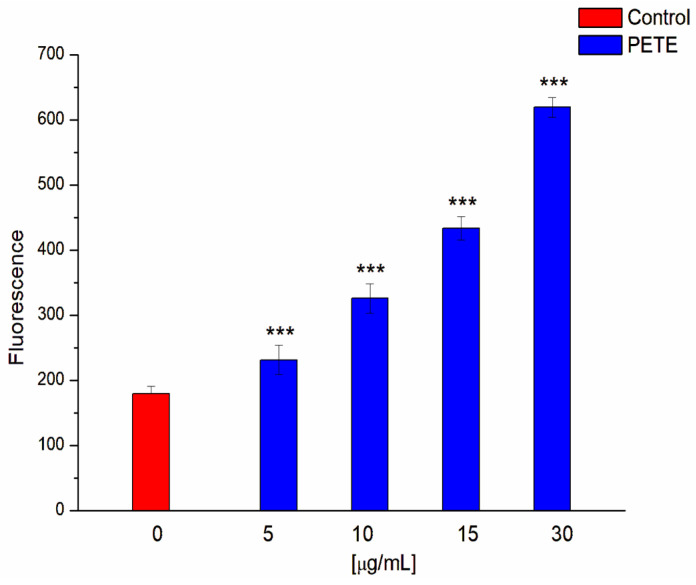
Formation of ROS in the presence of PETE in *S. aureus* cells. *** *p* < 0.001 vs. control (n = 5).

We have also investigated whether the extract, at concentrations of MIC and below, induces the formation of ROS in a concentration-dependent manner. Figure 7 shows that, indeed, the extract causes a doubling of the ROS content in bacteria at a half-MIC concentration and a tripling at a MIC concentration.

It is known that the antibacterial activity of polyphenols is realized at the level of the whole bacteria and at the level of released toxins, as well as at the level of target cells. In the case of whole bacteria, polyphenols can cause the modification or even damage of the membrane, induce an inhibition of energy metabolism and alter the expression of factors responsible for the survival function, including virulence factors and their secretion [63,64,65]. A number of flavonoids display a correlation between their antibacterial activity and increased ROS content in bacteria [66]. The formation of ROS in bacterial cells under the action of polyphenols can take different pathways and have different consequences. Low concentrations of ROS, primarily superoxide anion radicals (O_2_^.−^), can induce microbial population growth and activate mechanisms causing adaptive mutations. On the other hand, the oxidative stress induced by high concentrations of ROS leads to cell death [67,68]. Such a prominent pro-oxidant activity of flavonoids is primarily attributed to their influence on cell signaling and the regulation of certain genes in bacteria. For example, Zeng et al. showed that the licorice flavonoid licochalcone disrupts the arginine metabolism (mainly its synthesis that plays an important role in the urea cycle, NO and polyamine synthesis) and effectively inhibits the growth of *S. aureus* bacteria by inducing an increase in ROS levels [69]. It was found that flavonoids extracted from *Agrimonia pilosa Ledeb* showed antibacterial activity also through oxidative stress, which was accompanied by a decrease in the expression of the antioxidant enzyme genes (SodA, katA, TrxB) and by an increase in the expression of the gene PerR, which is a common H_2_O_2_-inducible transcription factor [70]. Similarly, the cytotoxic effect of catechin on *S. aureus* was mediated by ROS production and decreased activity of antioxidant enzymes like superoxide dismutase and catalase [71]. At the same time, it has been shown that catechins can induce ROS formation and DNA damage in methicillin-resistant *S. aureus* through autooxidation [72]. In our study, we have shown, for the first time, the relationship between *S. aureus* bacterial survival and ROS formation in the presence of an extract containing 90% ellagitannin. As noted above, ellagitannins exhibit the ability to form semiquinone and quinone oxidative products depending on pH, but also in the presence of oxidants [57], including the metals of variable valence [73], which, in turn, could consequently lead to the formation of ROS. In addition, tannins, due to their prominent protein binding properties, can fix to bacterial membrane proteins (and possibly to the electron transport chain proteins), which may lead to extracellular electron transfer [74] with the subsequent formation of ROS and, primarily, superoxide anions. Finally, the formation of superoxide anion radicals during the interaction of tannins with ATP synthase cannot be excluded. Such an interaction, leading to the inhibition of ATP synthesis, was shown for flavonoids and stilbenes [75,76].

## 4. Conclusions

In summary, our results indicate that the pomegranate extract exerts its action in a concentration-dependent manner yet differently depending on the cell model chosen. While, in erythrocytes, it acts mostly as an antioxidant and supports cell integrity, in cancer cells (HeLa), the extract most likely induces autophagic cell death via a ROS production potentiation and via mitochondrial dysfunction, even at lower concentrations than in red blood cells. The observed microbial cell (*S. aureus*) growth arrest and death can also be linked to robust ROS induction by the extract in the same concentration range. In vitro studies suggest that the pomegranate extract containing mainly ellagitannins has a strong antiradical action comparable to that of Trolox. The obtained data indicate the specificity of ROS production by the pomegranate extract depending on the type of cell, the concentration of the extract and the time of incubation. This specificity suggests a strong potential for the extract components as candidates in antioxidant and pro-oxidant therapy.

Polyphenols, being abundant constituents of a human daily diet, play a significant role in maintaining human health. One of the sources of polyphenols is pomegranate. Mainly the juice, but also the by-products, of pomegranate contain a broad spectrum of polyphenolic compounds, such as flavonoids, phenolic acids and tannins. These phytochemicals are generally known for their antioxidant action that prevents a number of pathologies connected to oxidative stress. The by-products of pomegranate, such as seed, leaves and peel, contain various tannins, with ellagitannins being the most abundant [4,51]. Ellagitannins display antioxidative properties as well; however, their ability to easily form radicals makes them a potential candidate as a prooxidant therapy against cancer cells and against pathological bacteria via the induction of ROS-mediated cell death. Further studies are needed to identify and isolate each component of the extract and to study their effects in different cell type populations, their mechanisms of action on the pathways of ROS induction and the consequences of ROS presence, as well as to investigate their cytotoxicity towards normal, non-malignant and host cells. It should be kept in mind, however, that the synergistic effect of constituents of the extract cannot be excluded, and so these studies should be conducted in comparison with the whole extract. We, and others, have recently shown that such whole extracts have much more pronounced effects than their isolated components [19]. Finally, a prospective direction in the study of ellagitannins from these extracts could be an investigation of their activity in photodynamic therapy, which is also connected to ROS induction [77].

## Figures and Tables

**Table 1:** Antiradical activity of PETE.

Radical	PETE	Trolox
	IC_50_ [µg/mL]	
DPPH	2.857 ± 0.223	4.051 ± 0.962
ABTS^+^	1.829 ± 0.091 *	2.365 ± 0.137
O_2_^−^	4.745 ± 0.279 ***	198.668 ± 2.065
NO^.^	21.914 ± 1.618 **	9.708 ± 1.492

Data represent analysis of three independent experiments. * *p* = 0.015, ** *p* = 0.005, *** *p* = 10^−5^ vs. Trolox, respectively.

**Table 2:** No acute effect of the PETE on the integrity and ROS level of erythrocytes.

PETE [µg/mL]	Hemolysis [%]	ROS [F/F_0_]
0	1.000 ± 0.254	1.000 ± 0.039
25	0.980 ± 0.462	1.000 ± 0.042
50	1.137 ± 0.497	1.008 ± 0.041
75	1.313 ± 0.573	1.005 ± 0.027
100	1.921 ± 0.362 *	1.003 ± 0.050

Data represent analysis of three independent experiments, * *p* = 0.034 vs. 0 µg/mL.

**Table 3:** Long-term (24 h) action of PETE on the integrity and ROS level of erythrocytes.

PETE [µg/mL]	Hemolysis [%]	ROS [F/F_0_]
0	2.925 ± 0.601	1.000 ± 0.120
25	3.457 ± 0.601	1.099 ± 0.062
50	4.137 ± 0.688	1.510 ± 0.029 *
75	4.048 ± 0.788	1.527 ± 0.042 *
100	4.373 ± 0.929	1.912 ± 0.133 **

Data represent analysis of three independent experiments. * *p* = 0.01, ** *p* = 0.006 vs. 0 µg/mL.

## Data Availability

The original contributions presented in the study are included in the article, further inquiries can be directed to the corresponding author.
